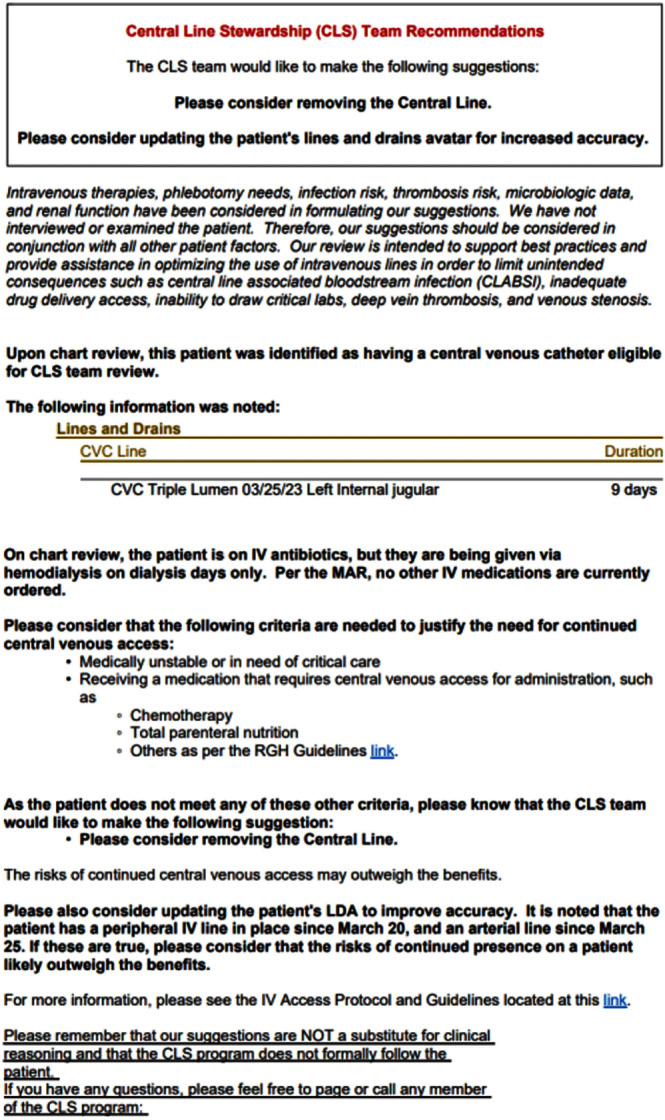# The Mechanics, Art, and Value of Central Line Stewardship

**DOI:** 10.1017/ash.2024.199

**Published:** 2024-09-16

**Authors:** Jennifer Gutowski, Maryrose Laguio-Vila, Gaby Razzouk, Anil Job, Chris Reynolds, Elizabeth Duxbury, Cunningham Kelly, Farhad Nasar, Emil Lesho

**Affiliations:** Rochester Regional Health; Rochester General Hospital

## Abstract

**Background:** Central venous catheter (CVC) utilization and central-line associated bloodstream infection (CLABSI) have increased nationwide. Busy providers can easily overlook the recommended practice of daily assessment of the ongoing indication for CVC. Prospective audit and feedback (PAF) is widely used and the gold standard for antibiotic stewardship programs (ASP), but reports involving PAF for device use are scarce. Therefore, we decided to evaluate the usefulness and feasibility of PAF for reducing device utilization and ultimately CLABSI rates at our 528-bed tertiary care hospital. **Methods:** A PAF-based Central Line Stewardship (CLS) initiative was launched in February 2023, with a team of hospitalists, infectious diseases physicians, health informatics, vascular access nurses, and infection preventionists. On business days, CVC line-lists were exported from the electronic medical record (EMR), into a REDCap (Research Electronic Data Capture) database for team members to evaluate, in real-time, all non-ICU CVCs in place for more >72 hours (Figures 1 and 2). For CVCs eligible for stewardship, CLS members placed Legal-approved note into the patient’s EMR, advising CVC removal or alternative IV access (Figure 3). CVCs continued to be audited until removal or patient discharge. Recommendation outcomes were tracked over the subsequent 72 hours for acceptance. Standardized Utilization Rates (SUR) and Infection Rates (SIR) were calculated and compared using the National Healthcare Safety Network. **Results:** Between February and August 2023, the CLS team reviewed 861 CVCs, representing 581 unique patient encounters, and made 622 recommendations. Recommendations to remove or replace the CVC represented 23.5% (146) of reviews, and 57% of these CVCs were removed within 3 days. 95% of removed lines had no adverse outcomes and did not require reinsertion. Hospital-wide CVC utilization decreased 18.7% from a SUR of 1.006 in the previous year, to 0.818 during the 7-month pilot period (p < 0 .001). In the 4 months following the pilot period, decreased CVC utilization was sustained with an SUR of 0.797. Non-ICU CLABSI SIR decreased from 1.282 in 2022 to 1.024 in 2023 (p=0.36). Average time physician required for CLS review approximated a 0.4 full-time equivalent a week. The intervention was well received, with requests for expansion to urinary catheters. **Conclusion:** CLS safely and significantly reduced device-utilization; directly via documentation and recommendation, and indirectly through increased awareness and the Hawthorne Effect. Examples of the “art” of CLS include when to leave a discoverable note and how to determine ongoing need for CVC in a fragile patient.

**Figure:**
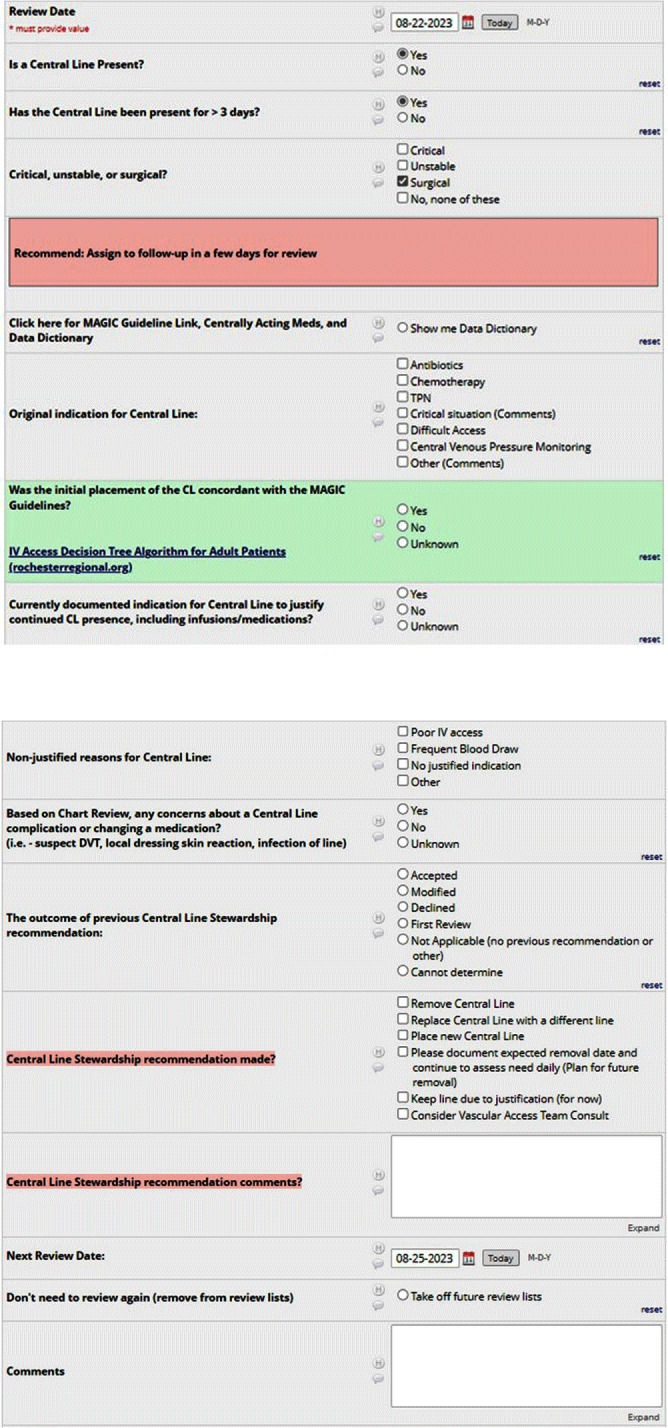

**Figure:**